# DNA fragments binding CTCF *in vitro *and *in vivo *are capable of blocking enhancer activity

**DOI:** 10.1186/1756-0500-5-178

**Published:** 2012-04-05

**Authors:** Dmitry A Didych, Elena S Kotova, Segey B Akopov, Lev G Nikolaev, Eugene D Sverdlov

**Affiliations:** 1 Shemyakin-Ovchinnikov Institute of Bioorganic Chemistry, Russian Academy of Sciences, 16/10 Miklukho-Maklaya, Moscow 117997, Russia

## Abstract

**Background:**

Earlier we identified ten 100-300-bp long CTCF-binding DNA fragments selected earlier from a 1-Mb human chromosome 19 region. Here the positive-negative selection technique was used to check the ability of CTCF-binding human genomic fragments to block enhancer-promoter interaction when inserted into the genome.

**Results:**

Ten CTCF-binding DNA fragments were inserted between the CMV enhancer and CMV minimal promoter driving the herpes simplex virus thymidine kinase (HSV*-tk*) gene in a vector expressing also the *neo*^R ^gene under a separate promoter. The constructs were then integrated into the genome of CHO cells, and the cells resistant to neomycin and ganciclovir (positive-negative selection) were picked up, and their DNAs were PCR analyzed to confirm the presence of the fragments between the enhancer and promoter in both orientations.

**Conclusions:**

We demonstrated that all sequences identified by their CTCF binding both *in vitro *and *in vivo *had enhancer-blocking activity when inserted between the CMV minimal promoter and enhancer in stably transfected CHO cells.

## Background

Spatial, temporal and tissue specific gene expression in mammals is largely determined by genomic cis-regulatory elements, such as promoters, enhancers, silencers, and insulators (for recent review, see [1,2]). A survey of about 1% of the human genome [3] indicated that the regulatory elements were more abundant in the genome than the genes they control and are mostly distal to the genes that they regulate.

While the number and positions of enhancer elements in the whole human genome can be determined with some certainty through P300 binding [4], the number and positions of most insulator elements are not known [2], and methods of their identification in mammals are sparse. Moreover, the definition of insulator is somewhat ambiguous--this term designates elements with enhancer-blocking or chromatin-bordering functions (reviewed in [5]) which are not interrelated at least in some cases [6,7]. In addition, the term "insulator" is sometimes used to designate the elements that bind the CTCF protein but have no proven enhancer-blocking or chromatin-bordering activity.

Two basic approaches have been proposed to identify many potential genomic insulators in one experiment. One approach is based on the ChIP-on-chip or ChIP-seq techniques with antibodies to known insulator-binding proteins, like CTCF or CP190 [8-10]. This approach can be used for the whole-genome analysis, but has a drawback that binding of a certain protein may be insufficient for insulator activity, which may result in many false positives. Another approach is based on a functional enhancer-blocking test in stably transfected cells [11,12] but is applicable to only relatively short (several megabases) genomic sequences.

It is well known that most mammalian insulators (with some exceptions reported [13-15]) bind CTCF (for review, see [16,17]). However, it was shown that CTCF has many other genomic functions apart from insulator [17].

Earlier we developed a positive-negative selection method allowing identification of insulators based on their ability to prevent promoter activation by enhancer when located between them [11]. We constructed a pPNT/EmP plasmid [11,12] containing the neomycin-resistance gene under control of the mouse phosphoglycerate kinase promoter (mPGK1) and the herpes simplex virus thymidine kinase (HSV-*tk*) gene under control of the CMV minimal promoter and CMV enhancer. HSV-*tk *catalyzes phosphorylation of ganciclovir to the monophosphate [18,19] which is further converted into the triphosphate by cellular enzymes and incorporated into growing DNA chain causing termination of replication and cell death [20,21].

The pPNT/EmP plasmid efficiently expresses the HSV*-tk *gene. However, after insertion into pPNT/EmP of a DNA fragment capable of blocking the interaction between the CMV promoter and enhancer, the HSV-*tk *expression in cells stably transfected with this plasmid gets significantly reduced and the cells become resistant to ganciclovir.

Using this principle, we developed a technique and used it for identification and mapping of 18 enhancer-blocking DNA elements within the *FXYD5-COX7A1 *region of human chromosome 19. This region contains more than 40 characterized genes with different expression profiles, and the data obtained allowed us to make conclusions on the mutual arrangement of enhancer-blocking sequences and genes and their possible functional interactions [11,12].

In this work, we studied the relationship between CTCF binding and enhancer-blocking activity of 10 CTCF-binding genomic fragments identified earlier in our laboratory [22]. Using a functional test described above, we demonstrated that all fragments which bind CTCF both *in vitro *and *in vivo *were capable of blocking activation of the CMV minimal promoter by the CMV enhancer in stably transfected CHO cells.

## Methods

### Basic protocols

Growth and transformation of *E. coli *cells, preparation of plasmid DNA, agarose gel electrophoresis, blot-hybridization and other standard manipulations were performed as described [23].

### Constructs

Ten *in vitro *CTCF-binding DNA fragments cloned previously in pGEM-T (Promega) [22] were cut out with *Xho *I and inserted in both orientations into pPNT/EmP [11] using *Sal *I site located between the CMV enhancer and minimal promoter.

A pPNT/mP plasmid containing the HSV-*tk *gene under control of the CMV minimal promoter and conferring resistance to neomycin and ganciclovir on transfected cells [11] was used as one of positive controls. Another positive control was a pPNT/E-sns-mP plasmid containing the sea urchin *Paracentrotus lividus *sns insulator between enhancer and promoter [24,25]. To prepare this construct, a pBS KS + plasmid, kindly provided by R. Melfi and G. Spinelli (University of Palermo, Italy), was cut with *Hin*d III and *Sma *I, treated with Klenow enzyme to fill in the sticky ends, and a ~300 bp fragment containing the sns insulator was isolated by agarose gel electrophoresis. The sns-containing fragment was inserted in both orientations into pPNT/EmP cut with *Sal *I and treated with Klenow enzyme.

A negative control pPNT/E-λ-mP plasmid was prepared by insertion of a lambda phage fragment PCR-amplified with *Xho *I site-containing primers ACTCGAGTCCGTGAGGTGAATGTG and ACTCGAGTAGTCGGCTCAACGTGG into *Sal*I-digested pPNT/EmP.

**Figure 1:**
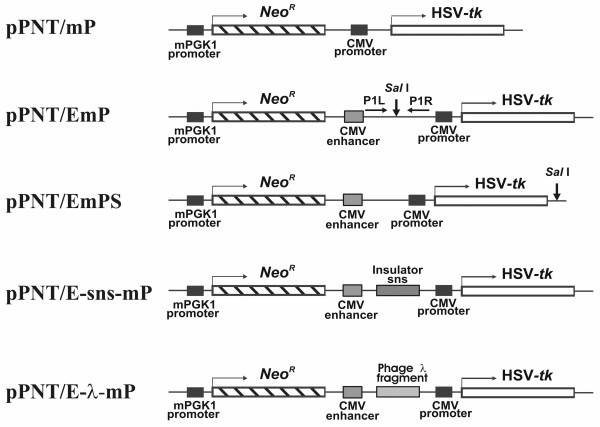
**Plasmid constructions used in this work**. mPGK-1-mouse phosphoglycerate kinase I promoter; *Neo^R^-E. coli *neomycin phosphotransferase gene; HSV-*tk*-herpes simplex virus thymidine kinase gene. Broken arrows indicate direction of transcription. For detailed description, see the text.

The constructs obtained are shown in Figure 1. Prior to electroporation, the constructs were linearized with *Eco*47 III (Fermentas).

To reveal possible silencer activity of CTCF-binding fragments, the *Sal *I recognition site of the pPNT/EmP plasmid was inactivated by *Sal *I digestion and filling in the sticky ends with Klenow enzyme followed by self-ligation. The plasmid was then cut with *Psp*E I (SibEnzyme), and double-stranded adapter (a hybrid of GTCACCAATTGTCGACGGATCC and GTGACGGATCCGTCGACAATTG) containing a *Sal *I site (underlined) was inserted 3' to the HSV-*tk *gene. The resulting plasmid (pPNT/EmPS, Figure 1) was used for insertion into the novel *Sal *I site of CTCF-binding fragments ## 3, 7 and 8. Prior to electroporation, the constructs were linearized with *Ssp *I (Fermentas).

### Cell culture and transfection

CHO-K1 cells (CCL-61, Chinese hamster ovary cells) were grown under conditions recommended by ATCC. Electroporation was performed using a Gene Pulser Xcell (BioRad) system as described previously [11], the transfected cells were inoculated into 5 ml of growth medium and incubated for 48 hours. The medium was then replaced with fresh culture medium supplemented with 500 ug/ml of G418 (Geneticin, Gibco-BRL), and the cells were cultured for 2 weeks in the presence of this antibiotic. An aliquot of the cell suspension was taken for genomic DNA isolation, and residual cells were cultured for 2 more weeks after addition of 4 or 10 uM of GANC (Sigma). The G418 and GANC resistant cells were then collected, and genomic DNA isolated using a Wizard Genomic DNA Purification Kit (Promega).

### PCR

The first stage of nested PCR was performed using a 20 ng genomic DNA template and primers matching the 5' and 3' flanks of the insertion site (*Sal *I). Structures of the primers are presented in Table 1. Primers P1L and P1R were used for pPNT/EmP based constructs and primers P2L and P2R - for pPNT/EmPS based constructs. The first stage product was diluted 4-fold and 1 ul thereof was used as a template for the second stage, where each individual internal primer was used in combination with either P1L or P1R in order to determine both the presence and orientation of the CTCF-binding fragments in the selected DNA (see Table 2). PCR was performed for 20-25 cycles at the first stage and 25-35 cycles at the second stage using the following profile: 94°/30 s; 60°/30 s; 72°/50 s.

**Table 1:** Sequences of PCR primers

Fragment ID	Primer ID	Sequence
1	1L	GTTTGTGACCTGTGCCCTTT
	1R	GAGGCCCCGACTCTTAACTC

2	2L	AGGTCCCTTCTCTCCCTGCT

3	3L	AATGATATTCCTCACGGCACT

4	4L	GCTCTGGGAAGAAACCACAG
	4R	GCAGGAGCAAGGTGAGATG

5	5L	GCTTTCTGACCCGCCTTT

6	6L	ATAGAGAAGCAGGGGGTGTG

	6R	TGCTGTTCCGTAATAACTTGCT

7	7L	CACTAATGAGAGACGCTGAGGA
	7R	GCTTCTGGAGGGTGTTTCTG

8	8L	CACTTTCTCCCACACTTCCA
	8R	CACCGTCCTCTGCCAACT

9	9R	AAGGCACTGGCATCCTGTCT

10	10L	GTACAGCCCTGGAGCAAGGAC

sns	snsL	ACTCGCAAACCTCAACACCT
	snsR	CAAAACTGGAATGGGGAAGA

Plasmid	P1L	GGATTTCCAAGTCTCCAGGGGAT
primers	P1R	ACCTCCCACCGTACACGCCT

Plasmid	P2L	CCGGACGAACTAAACCTGAC
primers	P2R	TGTAGGTACTCTGTTCTCACCCTTC

**Table 2:** Primer pairs for identification of CTCF-binding sequences in direct and reverse orientation

Fragment ID	Combination of primers*			
	
	Direct orientation		Reverse orientation	
	
	pPNT/EmP	pPNT/EmPS	pPNT/EmP	pPNT/EmPS
1	P1R, 1L (522)		P1R, 1R (286)	

2	P1R, 2L (268)		P1L, 2L (290)	

3	P1R, 3L (233)	P2R, 1L (319)	P1L, 3L (255)	

4	P1R, 4L (346)		P1R, 4R (317)	

5	P1R, 5L (307)		P1L, 5L (329)	

6	P1R, 6L (447)		P1R, 6R (320)	

7	P1R, 7L (292)	P2R, 7L (378)	P1R, 7R (305)	

8	P1R, 8L (341)		P1R, 8R (330)	P2R, 8L (416)

9	P1L, 9R (267)		P1R, 9R (245)	

10	P1R, 10L(557)		P1L, 10L (579)	

sns	P1R, snsL (354)		P1R, snsR (368)	

*Expected PCR product lengths (bp) are indicated in parentheses

Real-time quantitative PCR (qPCR) was performed using an MX3000P cycler (Stratagene) and qPCRmix-HS SYBR (Evrogen) in a 25 ul reaction volume for 40 cycles with the following profile: 95°C for 30 s, 65°C for 20 s, and 72°C for 30 s. The following primer pairs were used: GGCGTGGATAGCGGTTTGACT and GGCACTGTCCTCAGCGTCTCTC to reveal the pPNT/E-CTCF7-mP construct, GGCGTGGATAGCGGTTTGACT and ACGGATGGTGATGCCGAGAAC to reveal the pPNT/E-λ-mP construct, and ACTACGGCATCTCTGCCCCTTC and GGCACTGTCCTCAGCGTCTCTC to reveal the pPNT/EmPS-CTCF7 construct.

The relative DNA content was calculated according to the formula:

$$C=1/){\left(2\text{E}\right)}^{\text{N}}n^{-\text{N}}g$$

where C is the relative DNA content, E-efficiency of the primer pair, and N is the number of PCR cycles required to detect the target on templates isolated after Neo selection (N_n_) or after Neo and ganciclovir selection (N_g_).

The DNA contents of different constructs were normalized to that of pPNT/E-CTCF7-mP.

## Results

The following constructs were prepared from the pPNT/EmP plasmid (see Figure 1): (i) a pPNT/mP plasmid lacking the CMV enhancer; (ii) a pPNT/E-sns-mP plasmid, where the sns (silencing nucleoprotein structure) insulator from the sea urchin *Paracentrotus lividus *[24,25] was cloned in both orientations between the CMV enhancer and promoter. The sns insulator was kindly provided by G. Spinelli and R. Melfi (University of Palermo, Italy); (iii) a pPNT/E-λ-mP plasmid in which, instead of the sns insulator, a fragment of phage lambda DNA was placed between the CMV enhancer and promoter. In addition, the pPNT/EmPS control plasmid was prepared that allows to insert potential enhancer-blocker outside of the promoter-enhancer region thus making it possible to detect silencer activity of DNA fragments.

CHO cells were electroporated with these constructs followed by incubation for 48 h, addition of G418 and then positive selection for 12-14 days. In these conditions, non-electroporated control cells died within 7 days.

Once the selection was complete, an aliquot was taken from each selected sample for genomic DNA isolation, and the remaining cells were subjected to negative selection by addition of 4 or 10 micromoles of ganciclovir. At both ganciclovir concentrations, complete cell death was observed in samples transfected with the pPNT/EmP and pPNT/E-λ-mP plasmids, whereas in samples transfected with pPNT/mP and pPNT/E-sns-mP a significant portion of cells were resistant to the ganciclovir treatment. The partial cell death in these samples can be due to the activation of the HSV-*tk *promoter by endogenous cellular enhancers.

**Figure 2:**
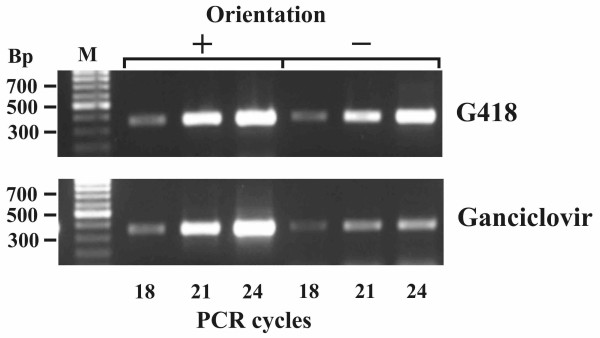
**PCR products obtained using a genomic DNA template from transfected CHO cells after positive (G418) and positive-negative (Ganciclovir) selection and primers specific to the sns insulator in direct (+) or reverse (-) orientation relative to the CMV minimal promoter**. M-DNA length marker (SibEnzyme).

After the positive-negative selection, genomic DNA was isolated from the pPNT/E-sns-mP transfected cells and used to determine the presence and orientation of the sns insulator inserts by PCR amplification (Figure 2). The primer pairs for amplification of the sns insulator in both orientations and their sequences are presented in Tables 1 and 2. As seen from Figure 2, the PCR produced DNA fragments of the expected lengths, which means that the HSV-*tk *expression in the transfected cells was suppressed or significantly reduced, and that the sns element in CHO cells was active as enhancer blocker in both orientations.

Therefore, the control experiments confirmed that the system developed could be used for selection of enhancer-blocking sequences.

### Enhancer-blocking activity of the CTCF-binding DNA fragments

To check the enhancer-blocking ability of ten CTCF-binding human genomic fragments identified earlier in our laboratory [22], we cloned them into pPNT/EmP between the cytomegalovirus enhancer and promoter. Since enhancer-blocking activity of some insulators was shown to be orientation-dependent [26], 20 plasmids containing the 10 fragments in different orientations relative to the promoter were prepared. For transfection, all 20 plasmids were linearized and pooled in equal amounts. The same amount of pPNT/E-sns-mP, containing the sns insulator in both orientations, was added to the pool as an internal control.

In this work we used the CHO cell line successfully employed earlier for selection of enhancer-blocking sequences [11,12]. These cells are advantageous for the selection because they are highly sensitive to ganciclovir and can be efficiently transfected by electroporation. Moreover, it was shown that insulators from the genome of one species can maintain their activity in the cells of other species. In particular, the 5'-HS4 chicken beta-globin insulator is active in human K562 cells [27], and the sea urchin sns insulator can block enhancer-promoter interactions in human osteosarcoma cells U2-OS and human lung adenocarcinoma cells H1299 [24]. It was also shown that human CTCF can bind to corresponding sites in the mouse genome [28].

CHO cells were transfected with the plasmid pool by electroporation using conditions established previously to provide integration of a single plasmid copy into the cell genome [29], and then subjected to positive-negative selection as described above. The survived cells were used to isolate genomic DNA.

**Figure 3:**
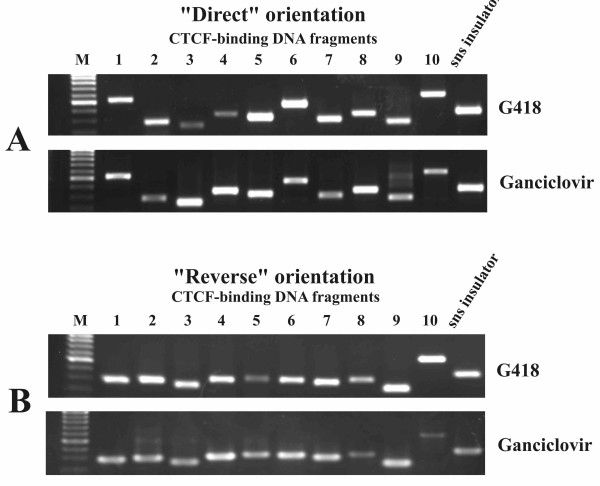
**PCR products obtained using a genomic DNA template from transfected CHO cells after positive (G418) and positive-negative (Ganciclovir) selection and primers specific to 10 CTCF-binding DNA fragments**. A-"direct", and B-"reverse" orientation of the fragments relative to the CMV minimal promoter. M-DNA length marker (SibEnzyme).

The genomic DNA was used as a template for nested PCR. At the first stage, the fragments located between the CMV promoter and enhancer were amplified with primers P1L and P1R (Figure 1). The PCR product contained a mixture of selected CTCF-binding fragments flanked by short fragments of the pPNT/EmP DNA. This mixture was used as a template for the second PCR round with internal primers specific for each CTCF-binding fragment and the control sns insulator (Table 2). Each individual internal primer was used in combination with either P1L or P1R in order to determine both the presence and orientation of the CTCF-binding fragments in the selected DNA. The results of nested PCR are presented in Figure 3. As seen from Figure 3A,B (upper panels), all 10 CTCF-binding fragments and the control sns insulator were present in the genomic DNA after G418 selection suggesting that the corresponding constructs were inserted into the cellular genome. The same fragments were revealed also after selection with 10 uM (Figure 3A,B, lower panels) or 4 uM ganciclovir (not shown). Therefore, it can be concluded that all 10 fragments which bind CTCF *in vitro *make the cells resistant to ganciclovir when placed between enhancer and promoter.

It should be noted that, apart from insulators, other regulatory elements, silencers, might also confer resistance to ganciclovir. However, silencers are known to suppress promoter activity irrespective of their position relative to the promoter [30]. Therefore, in the constructs used, silencers would suppress also the activity of the neomycin phosphotransferase promoter, and the transfected cells would not survive positive selection [11]. Nevertheless, we checked possible silencer activity of three (## 3, 7 and 8) CTCF-binding fragments by cloning them into the pPNT/EmPS plasmid 3' to the minimal promoter (Figure 1). The cells transfected with these constructs died at the negative selection stage suggesting that the fragments analyzed did not suppress the CMV promoter and hence did not possess silencer activity. Thus, these fragments could block the enhancer action only when placed between promoter and enhancer.

**Figure 4:**
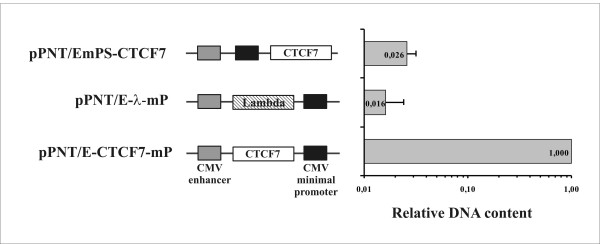
**Relative DNA content of three constructs: pPNT/E-CTCF7-mP that contains the CTCF7 fragment between the promoter and enhancer, pPNT/EmpS-CTCF7 that contains the CTCF7 fragment outside the promoter-enhancer pair, and pPNT/E-l-mp--the control construct with a lambda DNA fragment inserted between the promoter and enhancer in genomic DNA of CHO cells after positive-negative selection**. The relative content was estimated by real-time PCR based on the difference between the number of PCR cycles required to detect the target (for detail, see Methods).

To quantitatively estimate the efficiency of the CTCF binding sequences selection, we pooled together three constructs, namely pPNT/EmP with the CTCF7 fragment inserted between the promoter and enhancer, and two controls-pPNT/EmPS with the CTCF7 fragment inserted outside the promoter-enhancer pair and pPNTE-λ-mP with the lambda phage fragment inserted between the promoter and enhancer. This equimolar mixture was subjected to positive-negative selection procedure described above. Genomic DNAs were then isolated from Neo and ganciclovir resistant cells and used as templates for real-time quantitative PCR. The results are presented in Figure 4.

As seen from the figure, the relative DNA content (measured as described in Methods) of the control constructs was 30-40 times lower than that of the CTCF binding fragments. These data support high efficiency of the selection procedure and open up the opportunity for quantitative measurement of the enhancer-blocking effects.

## Discussion

It can be therefore concluded that all 10 fragments from the *FXYD5-COX7A1 *region that bind CTCF *in vitro *make cells resistant to ganciclovir when inserted between enhancer and promoter, i.e. have enhancer-blocking (insulator) activity irrespective of their orientation relative to the promoter. In addition, the enhancer-blocking activity of the sea urchin sns insulator is also independent on its orientation, supporting previous findings [31]. Whereas many consider insulators to be orientation-dependent, there are multiple examples of orientation-independent insulators [32-35].

Earlier we demonstrated [11] that 6 out of 8 sequences identified by their ability to block enhancer-promoter interactions were capable of binding CTCF both *in vitro *and *in vivo*. Here, we present the evidence that all sequences under study identified by their CTCF binding have an enhancer-blocking activity.

The CTCF binding and enhancer-blocking potential are not necessarily interrelated, and at least enhancer-blocking ability can exist without CTCF binding [13-15]. This observation is in line with *Drosophila *data [36] showing that different subclasses of insulators bind different proteins, such as dCTCF, GATA, Su(Hw), or BEAF, and only part of insulators bind dCTCF. On the other hand, the large number of CTCF binding sites in the genome suggests a wide CTCF versatility far exceeding just insulator function and including context-dependent promoter activation/repression, hormone-responsive silencing, genomic imprinting, and long-range chromatin interactions (reviewed in [17]).

Recent genome-wide studies on CTCF occupancy in different cell types shed light on general characteristics of CTCF binding sites distribution with respect to positions of genes in the genome (see references in Table 3). Table 3 shows that that there are cell-type specific differences in occupancy, but it is still unclear whether they are functionally significant or merely due to differences in computational and experimental procedures used [37]. Although cell-type specificity of CTCF occupancy is in apparent contradiction with the conclusion that most insulator elements are not specific to individual cell types [2], it is in line with the observation that occupancy of CTCF sites is dependent on their DNA methylation status (see [38] for review).

**Table 3:** Summary of the number and location of human potential enhancer-blocking elements

Cells	Potential insulators (CTCF binding and enhancer- blocking sites)	Intergenic	Intronic or exonic	Within ± 2 kb from promoter	Technique used	**Ref**.
IMR90 human fibroblasts	13804	46%	34%	20%	ChIP-chip	[10]

Resting human CD4+T-cells	28661	49%	36%	15%	ChIP-Seq	[9,39]

HeLa	19308	56%	37%	7%	ChIP-Seq	[9]

Jurkat	19572	55%	36%	9%	ChIP-Seq	[9]

Mouse embryonic stem cells	39609	N/D	N/D	N/D	ChIP-Seq	[40]

HeLa/CHO	28 (84000/genome)	46%	36%	18%	Positive- negative selection	[11,12]

N/D-no data

The negative-positive selection data on enhancer-blocking sequences obtained in this study, together with those reported by us previously [11,12], are summarized in Table 3 along with the ChIP-chip and ChIP-seq data available to-date. While the distribution of potential enhancer-blocking sequences among different genomic regions examined by different techniques is very similar, the number of the enhancer-blocking sites in the genome calculated by extrapolation of the number within a 1000-kb genomic region found by us is considerably higher than genome-wide evaluations. It is difficult now to ascertain the reason for this discrepancy. For instance, negative-positive selection might detect additional enhancer-blocking elements which do not bind CTCF.

## Conclusions

We would like to note that although our data, as well as data of other authors, provide more or less comprehensive structural information; a great challenge is to translate this information into the language of insulator function. This challenge is to a large extent due to various mechanisms of action of different insulators and cannot be resolved by a genome-wide approach. It demands thorough analyses of candidate insulators to reveal all components of the regulatory networks that involve these regulatory elements. Proper techniques for such analyses, as e.g. 3 C, 4 C and 5 C, are already being intensively developed.

## Competing interests

The authors declare that they have no competing interests.

## Authors' contributions

SA, DD and EK designed and prepared the constructs, SA and DD performed the transfection experiments. LN, SA and DD participate in data interpretation; LN was responsible for writing of the manuscript. EDS conceived and coordinate the study and participate in drafting of the manuscript. All authors read and approved the final text.
